# Role of Vaccination Strategies to Host-Pathogen Dynamics in Social Interactions

**DOI:** 10.3390/e26090739

**Published:** 2024-08-30

**Authors:** Marlon Nunes Gonzaga, Marcelo Martins de Oliveira, Allbens Picardi Faria Atman

**Affiliations:** 1 Programa de Pós-Graduação em Modelagam Matemática e Computacional, Centro Federal de Educação Tecnológica de Minas Gerais—CEFET-MG, Ave. Amazonas, 7675-Nova Gameleira, Belo Horizonte 30510-000, MG, Brazil; atman@cefetmg.br; 2 Departamento de Estatística, Física e Matemática, Universidade Federal de São João del-Rei-UFSJ, Ouro Branco 36495-000, MG, Brazil; mmdeoliveira@ufsj.edu.br; 3 Departamento de Física, Centro Federal de Educação Tecnológica de Minas Gerais—CEFET-MG, Ave. Amazonas, 7675-Nova Gameleira, Belo Horizonte 30510-000, MG, Brazil; 4 National Institute of Science and Technology for Complex Systems—CEFET-MG, Belo Horizonte 30510-000, MG, Brazil

**Keywords:** efficient vaccination strategies, host-pathogen interactions, agent-based modeling, complex systems

## Abstract

This study presents extended Immunity Agent-Based Model (IABM) simulations to evaluate vaccination strategies in controlling the spread of infectious diseases. The application of IABM in the analysis of vaccination configurations is innovative, as a vaccinated individual can be infected depending on how their immune system acts against the invading pathogen, without a pre-established infection rate. Analysis at the microscopic level demonstrates the impact of vaccination on individual immune responses and infection outcomes, providing a more realistic representation of how the humoral response caused by vaccination affects the individual’s immune defense. At the macroscopic level, the effects of different population-wide vaccination strategies are explored, including random vaccination, targeted vaccination of specific demographic groups, and spatially focused vaccination. The results indicate that increased vaccination rates are correlated with decreased infection and mortality rates, highlighting the importance of achieving herd immunity. Furthermore, strategies focused on vulnerable populations or densely populated regions prove to be more effective in reducing disease transmission compared to randomly distributed vaccination. The results presented in this work show that vaccination strategies focused on highly crowded regions are more efficient in controlling epidemics and outbreaks. Results suggest that applying vaccination only in the densest region resulted in the suppression of infection in that region, with less intense viral spread in areas with lower population densities. Strategies focused on specific regions, in addition to being more efficient in reducing the number of infected and dead people, reduce costs related to transportation, storage, and distribution of doses compared to the random vaccination strategy. Considering that, despite scientific efforts to consolidate the use of mass vaccination, the accessibility, affordability, and acceptability of vaccines are problems that persist, investing in the study of strategies that mitigate such issues is crucial in the development and application of government policies that make immunization systems more efficient and robust.

## 1. Introduction

In the 18th century, Edward Jenner was responsible for conducting an experiment that would change society, probably, forever. In 1796, Edward Jenner observed that people who lived with cows infected by cowpox did not get sick due to smallpox. After this observation, he inoculated a small dose of cowpox in an eight-year-old boy (James Phipps) who got sick and developed a mild form of the disease. After his recovery, Edward Jenner took a fatal dose of smallpox and introduced it to the child. James Phipps became immune after the contact with cowpox and did not develop smallpox [1,2].

Smallpox vaccination paved the way for several other important vaccine developments and applications throughout history. The majority of developed vaccines work through the infusion of weakened pathogens into the organism, acting by destroying or weakening pathogens. The presence of these pathogens stimulates the immune system to produce antibodies that generate a preventive immunity response against a particular disease [3]. Recent epidemiological events have shown that the application of large quantities of vaccine doses requires a very efficient vaccination strategy. For instance, against Rubella, the vaccination of teenage girls in the United Kingdom from 1971 to 1988 emphasized the reduction of susceptible individuals, ensuring that the maximum amount of women who acquired previous immunity before the reproductive age. Another strategy promoted the vaccination of two-year-old boys and girls, leading to a decrease in positive cases of Rubella [4].

More recently, the World population faced the pandemic of SARS-CoV-2, which caused profound changes in several aspects. Despite the virus’s fast spread and damage, the development of vaccines brought the hope of better days at record speed. During the COVID-19 pandemic, vaccine development and distribution became crucial for limiting disease transmission [5]. Four months after the community spread of the virus was announced, a SARS-CoV-2 vaccine first generation was developed in silico. In July 2020, Moderna and Pfizer started large-scale phase II and III COVID-19 vaccine trials. In January 2021, just over a year after the first case of contamination by the new coronavirus, India approved the first DNA vaccine against SARS-CoV-2. In December 2022, there were 50 COVID-19 vaccine candidates approved by at least one country in the world [6]. Knowing that the world is increasingly connected and the global population is growing, investing in immunization strategies is crucial. In this context, it is also essential to consider the population composition and the structure of social networks [7].

With the development of science, mathematical and computational models have been consolidated as efficient methods to estimate complex processes, such as infection dynamics [8,9,10] or interactions between the human immune system and a pathogen [11], for instance. One of these tools is Agent-based modeling (ABM), a powerful and very adaptable approach for exploring complex systems and reproducing the manifestations of emergent phenomena. Different empirical complex systems are studied using ABM [12,13,14], including the development of models involving vaccination strategies. In a study using ABM and analysis of empirical data [15], authors studied scenarios with anti-vaccination (AV) movements considering contacts with medical practitioners and public vaccination campaigns, interpersonal communication, and the digital world’s influence. Another study combines genetic algorithms and ABM in a model that provides parameters for strategies of vaccination considering different population groups [16]. Recent works use empirical data to analyze epidemic dynamics, focusing, for example, on the experimental quantification of social contacts, which demonstrated that interpersonal contacts have a gamma distribution [17]. In a novel study [18], the authors merged ABM with a set of kinetic equations that describe the temporal evolution of the distribution of the number of agent contacts. The macroscopic dynamics of the spread of a pandemic are governed by the rules of the SIR model, enabling the development of a system that explains the relationship between the number of interpersonal contacts and the spread of an epidemic. Additionally, this system was applied to analyze the efficiency of applying non-pharmacological measures in the context of viral spread [19]. It is of the utmost importance to search for advanced models and techniques involving the vaccination process since challenges such as development, affordability, accessibility, and acceptability persist [4,20].

The present work employs a recently introduced model, the IABM (Immunity Agent-Based Model) [21] to describe and analyze distinct vaccination scenarios, showing how infection curves vary in response to a vaccination process carried out before the occurrence of a new outbreak. The original IABM addresses infection behavior at two levels: the individual level (microscale), which encompasses innate and humoral immune responses, and the social level (macroscale), comprised of pathogen transmission within a community. In this work, we add vaccinated individuals who exhibit a boost in their level of antibodies and study the dynamics between viral load, innate immune system response, and the work carried out by antibodies forming a microscopic dynamic within the model. Macroscopically, interpersonal interactions and epidemiological state transitions based on the SVEIR (*Susceptible-Vaccinated-Exposed-Infected-Recovered*) [22] model guide pathogen transmission between agents in the lattice. By coupling this microscopic host-pathogen dynamic with macroscopic dynamics using the SVEIR model, this approach represents epidemic dynamics similar to studies of other types, such as those combining interpersonal contact models and compartmental epidemiological models [18,19], for example.

The possibilities of reinfection were not considered in this study, since the reinfection rates of diseases such as COVID-19 and influenza, both diseases transmitted mainly by airways, are relatively low and within a short period of time, considering the circulation of the same variant of the virus [23,24,25]. We studied a type of vaccine that does not fully guarantee the individual’s immunity but reduces the chance of infection and the severity of symptoms.

The remainder of this paper is organized as follows. In Section 2, we provide a brief review of the main characteristics of the IABM. The methodology behind the model’s design is described in Section 3, while Section 4 presents the obtained results and an in-depth discussion of these findings. Finally, Section 5 encapsulates the conclusions drawn from our study.

## 2. The Immunity Agent-Based Model (IABM)

**Figure 1:**
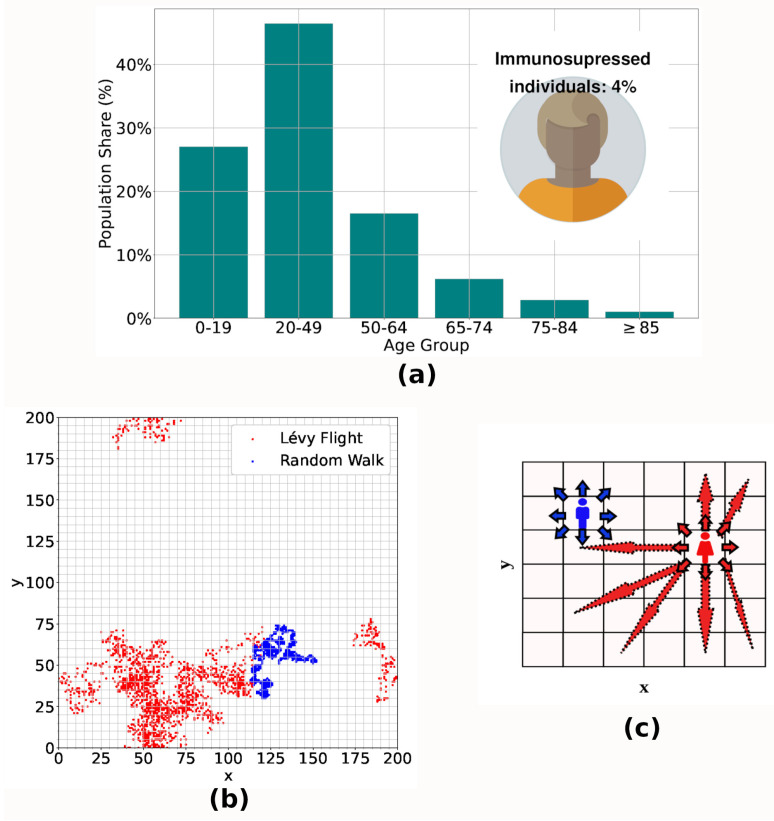
(**a**) Population age structure adopted in the model, based on an empirical population [26]. Immunosuppressed individuals represent 4% [27] of the total population. (**b**) shows the typical displacement patterns of agents moving as random walkers (blue) and performing Lévy Flights (red). Due to the exponential distribution of step lengths, agents performing Lévy Flights can reach more sites on the lattice. In (**c**), we see an artistic representation of these types of movements, highlighting the jumps characteristic of Lévy Flights (red individual) and the short steps typical of random walkers (blue individual).

The Immunity Agent-Based Model (IABM), detailed in our previous publication [21], is a versatile tool for investigating airborne diseases caused by different pathogens. As in the previous work, we study the spread of the virus, calibrating the model parameters considering COVID-19 characteristics (it is important to say that the calibration was carried out considering the original model, but the objective of this work is to provide analyses that support the definition of vaccination strategies that could be applied in immunization processes for different airborne diseases). The model stratifies the population by age, categorizing individuals into groups of 0–19, 20–49, 50–64, 65–74, 75–84, and older than 85, whose number of individuals in each range is based on the age pyramid of a real population [26], as seen in Figure 1a. Additionally, 4% [27] of the population represent the group of immunosuppressed individuals, whose innate and humoral response efficiencies are reduced in relation to an immunocompetent individual.

The model operates on two distinct levels: a microscopic level, focusing on interactions between immune system defense cells and invading pathogens, and a macroscopic level, where agents make social interactions, promoting airborne pathogen transmission. At this level, Epidemiological modeling of epidemiological state transitions is based on the SEIR (*Susceptible-Exposed-Infected-Recovered*) compartmental model. Our findings indicate the promising utility of the IABM, particularly in exploring vaccination scenarios. Unlike conventional models with predefined transition rates, the IABM captures the dynamic nature of transitions between epidemiological states, influenced by the intricate interplay between defense cells and pathogens. This feature results in a highly heterogeneous representation of real-world scenarios, enabling the simulation and analysis of vaccination outcomes, for example.

In the IABM, the leukocytes count [28] forms the basis of the innate response (ψ(t)) of each individual (agent) and immunocompromised individuals (individuals over 74 years of age and immunosuppressed individuals) present a reduction in the efficiency of their immune system compared to immunocompetent individuals. During the spread of the virus, individuals inhale a portion of the viral particles expelled by infected individuals. After contact with the virus, the innate immune system responds by capturing a viral particle with a scaled probability $p=(1-\alpha_{\psi }\cdot I_{age})\cdot u$, where u∼U[a,b] is a random variable uniformly distributed in the ranges [a,b] with $\alpha_{\psi }=4.5\times 10^{-3}$, $a=1.0\times 10^{-3}$ and $b=6.0\times 10^{-2}$. These probability and parameter values were defined in order to represent the senescence effect of the immune system, with its efficiency decreasing as the age ($I_{age}$) of the individual increases, based on the probability of defense system cells detecting and destroying antigens [29].

A time $\tau_{\eta }$ [30] was previously set for each agent, and this time signals the commencement of antibody production, triggering the humoral response. Temporal variation in the amount of antibodies is ${\left(\eta \right)}_{t}=\left(1-\delta_{\eta }\right)\cdot {\left(\eta \right)}_{t-1}+\lambda_{\phi }\cdot {\left(\phi \right)}_{t-1}-\lambda_{\eta }{\left(\eta \right)}_{t-1}+\alpha_{\eta }{\left(\psi \right)}_{t-1}$. $\lambda_{\phi }$ is the rate of antibody production triggered by the presence of the antigen in the organism, forming the acute immune response. The long-lasting production of antibodies is made with a rate $\alpha_{\eta }$. The viral particle is eliminated by antibodies with a rate $\lambda_{\eta }$, with the subsequent reduction in antibody levels due to the opsonization process [31].

## 3. Methods

This section presents a resume of the methodology of designing the IABM [21] model and a description of the vaccination process modeling. We designed an Agent-Based Model (ABM) and conducted Monte Carlo simulations on a square lattice with a linear extension L = 200 and periodic boundary conditions. Each site of the lattice has 10 m^2^ of area and 2990 agents move across the lattice as random walkers or Lévy Flights [32]. The typical displacement patterns are shown in Figure 1b. A total of 20% [33] of the agents were predefined to move by performing Lévy flights, with step sizes X=(−1/λ)·lnU (with λ=1.0 and U∼Uniform(0,1)) to represent individuals with greater displacement. The agents’s spatial distribution follows the Zipf law [34] and a greater statistical weight associated with the probability of shift is assigned to sites with more occupancy, ensuring that agents are more likely to move if they are in sparsely occupied regions.

### 3.1. Modeling the Vaccination Process

The vaccination process is conducted a priori: Upon the initiation of virus transmission, a subset of individuals has already been vaccinated, thereby establishing a humoral response in these people. Vaccinated individuals start the dynamic process with a preexisting level of antibodies, whose production adheres to the circadian cycle (similar to the innate response [21]). Here, the humoral response generated by vaccination mirrors that provoked by natural infection, since the natural and vaccine-induced immunity are equivalent for the protection against SARS-CoV-2 [35].

We consider the strategies described in Table 1. In Strategy 2, we will focus on individuals who are at greater risk of dying if infected, that is, individuals over 74 years of age and immunosuppressed, constituting the group called immunocompromised. Strategies 3 and 4 deal with the spatial distribution of vaccinated individuals. Strategy 1 considers random vaccination with a random distribution of doses.

The efficiency of the vaccine is linked to the capacity of antibodies to neutralize the viral particles. As previously mentioned, the natural production of antibodies after natural infection depends on the innate response as ${\left(\eta \right)}_{t}\to \alpha_{\eta }\cdot {\left(\psi \right)}_{t-1}$. For a vaccinated individual, the induced production of antibodies is ${\left(\eta \right)}_{t}\to \alpha_{vac}\cdot {\left(\psi \right)}_{t-1}$, with $\alpha_{vac}=\alpha_{\eta }$. When a vaccinated individual becomes infected, the long-lasting production of antibodies is made with a rate $\alpha_{vac}+\alpha_{\eta }$. The results presented here were obtained using $\alpha_{\eta }=1.0$. Notice that as $\alpha_{vac}$ is increased, the efficacy of the vaccine increases, and vice versa.

### 3.2. Epidemiological Modeling

**Figure 2:**
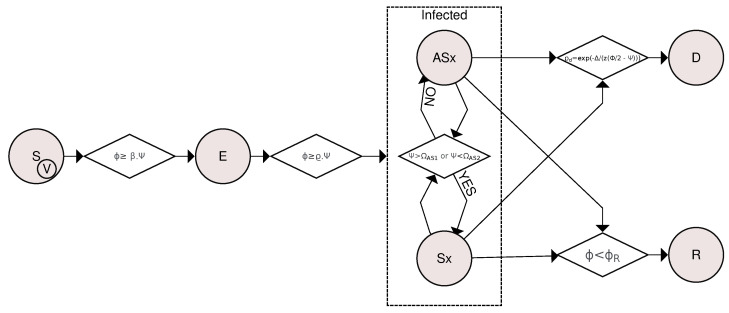
Flowchart for compartmental transitions based on the SVEIR (*Susceptible-Vaccinated-Exposed-Infected-Recovered*) model. The transitions are determined based on the viral load and the organism’s response to the pathogen through both innate and humoral immune mechanisms. A susceptible or vaccinated individual is exposed if their viral load ϕ equals or exceeds β·ψ, where β is a rate and ψ is the level of the individual’s innate response. If ϕ≥ϱ·ψ for at least 12 h straight, an exposed individual becomes infected. The *Asymptomatic*⟷*Symptomatic* transition depends on the impact of viral activity on the innate immune response, leading to symptoms if the immune response is exacerbated ($\psi >\Omega_{AS1}$) or if the immune system is weakened ($\psi <\Omega_{AS2}$). Otherwise, the individual is considered asymptomatic. The likelihood of death in symptomatic and symptomatic individuals is represented as $p_{d}=\exp\left(-\Delta /(z\cdot (\Phi /2-\psi ))\right)$. Recovery occurs if the viral load ϕ is less than $\phi_{R}$.

Figure 2 shows a flowchart for compartmental transitions based on the SVEIR model. The transitions are determined based on the viral load and the organism’s response to the pathogen through both innate and humoral immune mechanisms. A susceptible (S) or vaccinated (V) individual becomes exposed (E) if his or her viral load ϕ is greater than or equal to β·ψ. If ϕ≥ϱ·ψ for at least 12 h straight after an exposed individual becomes infected, where β and ϱ are transition rates. The Asymptomatic(Ax)↔Symptomatic(Sx) transition was based on how the innate response is affected by viral activity, becoming symptomatic if $\psi >\Omega_{AS1}$ or $\psi <\Omega_{AS2}$ and becoming asymptomatic otherwise. Asymptomatic and symptomatic individuals have a likelihood of death represented by $p_{d}=\exp\left(-\Delta /\left(z\cdot \left(\Phi /2-\psi \right)\right)\right)$ or recovery if his or her viral load ϕ is less than the threshold $\phi_{R}$. The difference between viral load and innate response is given by Δ=ϕ−ψ and *z* is a rescaled parameter.

## 4. Results and Discussion

This section reports and discusses the results of extended simulations of the IABM, considering distinct vaccination strategies. Similar to the original work [21], the simulations start with three infected individuals with an initial viral load, each with an initial viral load following a normal distribution N(μ,σ)=N(80.0,1.0). The parameters μ and σ were defined so that the dynamics begin with infected individuals having viral loads close to their level of innate immune response.

**Figure 3:**
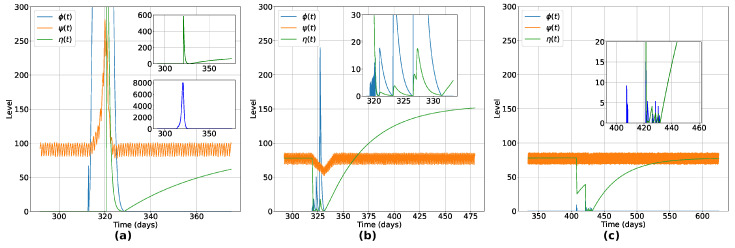
Typical variation of Innate Response (ψ(t)), Viral Load (ϕ(t)) and Humoral Response (η(t)) for three distinct situations: (**a**) is an unvaccinated individual that recovered after infection, (**b**) is a vaccinated individual that got sick and recovered and (**c**) a vaccinated individual that did not get sick. We see that, in (**a**), an unvaccinated individual can reach much higher viral load levels when compared to a vaccinated individual, leading to the appearance of symptoms. We observe in (**b**) a decrease in the innate response in the acute phase of the infection, with a rise-and-fall dynamic between the viral load and the levels of humoral immunity of the vaccinated individual who ends up being infected by the virus. After recovery, the individual presented a reinforced humoral response, as observed in some real cases of people infected with SARS-CoV-2 [37]. In (**c**), we see the typical behavior of a vaccinated individual who, even after having contact with the virus, the immunity induced by the vaccine is sufficient to prevent them from becoming sick, destroying the viral particles and allowing the individual to remain uninfected.

We begin by analyzing the effects of the vaccination at the microscopic (individual) level. In Figure 3, we show the variation of the innate response level (ψ(t)), viral load level (ϕ(t)), and humoral immunity level (η(t)) for three agents, to illustrate the typical behaviors observed in all recovered individuals. Figure 3a shows the typical variations for an unvaccinated individual that recovered after infection. Notice that the viral load of this individual reaches an impressively high level when compared with a vaccinated individual (Figure 3c), leading this individual to present symptoms during the active phase of the disease (due to the enhancement of the innate immune response [36]). In the second panel (Figure 3b), a vaccinated individual became sick after contact with the virus. It is observed that there is a decline in the innate response during the acute phase of the infection, with a rise and fall dynamic between viral load and humoral immunity levels. After recovery, the individual presented a reinforced humoral response, as observed in some real cases of people infected with SARS-CoV-2 [37]. In Figure 3c, typical behavior of a vaccinated individual is illustrated. Even after having contact with the virus, the vaccine-induced immunity is enough to prevent the individual from getting sick, destroying the viral particle, allowing the the individual to remain uninfected. In this case, the presence of the virus was eradicated before the minimal impact on their organism due to viral activity, with no reinforcement of the humoral immunity due to natural infection.

**Figure 4:**
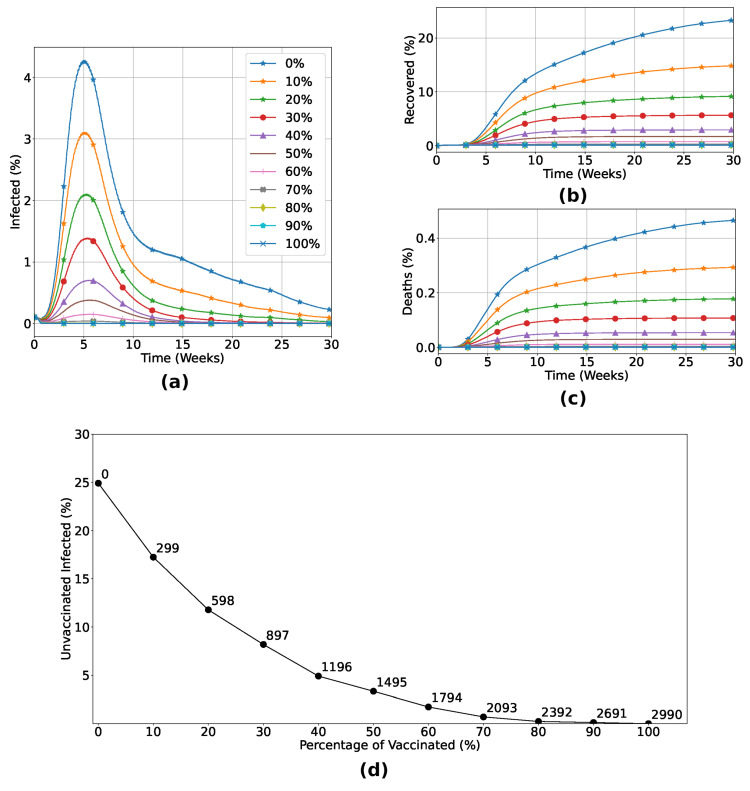
Percentage of infected (**a**), recovered (**b**) and dead (**c**) individuals considering Strategy 1. Panel (**d**) shows the variation in the percentage of unvaccinated individuals who become infected in relation to the increase in the number of vaccinated individuals. The number of doses is highlighted at each point of the graph. (**a**–**c**) certify that the vaccination process inserting a non-zero level of humoral immunity induces the reduction of infected and dead individuals, eliminating the outbreak with rates of vaccination greater than 70%. Panel (**d**) shows the herd immunity effect, with exponential reduction of infection of unvaccinated individuals while the number of vaccinated individuals increases. In particular, for the vaccine efficiency used, with 70% of the total individuals vaccinated, less than 2% of the unvaccinated group became infected, highlighting the indirect protection due to the vaccinated individuals in the lattice.

Now, we turn our attention to the macroscopic (population) level. In this first analysis, we consider that a fraction of the population is vaccinated at random, regardless of its spatial location, age, or immunological capabilities. Our results are detailed in Figure 4. As expected, the number of infected individuals decreases when the fraction of vaccinated individuals increases, resulting in a minor death rate, as shown in Figure 4a–c. In particular, for the vaccine efficiency used, the number of dead individuals vanishes for vaccination rates above 70%. This is a consequence of the herd immunity effect, as can be seen from the data in Figure 4d, where we observe an exponential reduction of infection of unvaccinated individuals while the number of vaccinated individuals increases. With 70% of the total individuals vaccinated, less than 2% of the unvaccinated group became infected, highlighting the indirect protection due to the vaccinated individuals in the lattice.

Until this point, we have assumed that vaccines are available to all individuals. However, for a new emerging disease, there may be a limited availability of doses due to the developing and manufacturing time and/or costs. In such cases, when only a small fraction of the population will be inoculated, an important question that arises is the following: which strategies, taking into account the heterogeneity of the population (due to age group, immunocompetence, and a greater number of daily contacts in denser regions), will be more successful in reducing the number of infected individuals and deaths? In the next subsection, we will apply the IABM to investigate three additional strategies.

**Figure 5:**
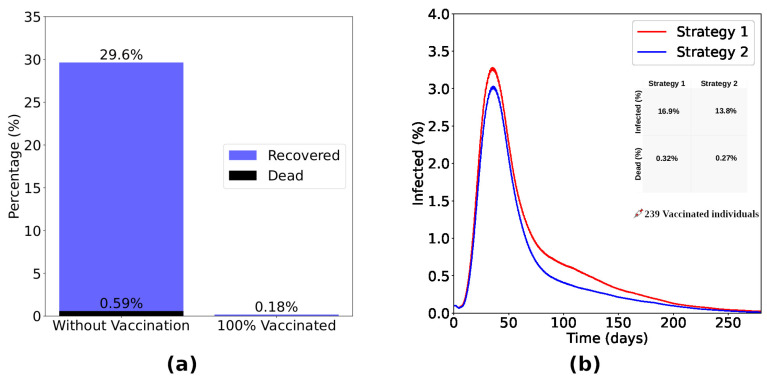
The bar chart shown in (**a**) presents the difference between a scenario without vaccination and a scenario considering the vaccination of 100% of immunocompromised individuals (Strategy 2). Without vaccination, 29.6% of the immunocompromised became infected, with the death of 0.59% of the same group. Vaccinating 100% of these individuals, the percentage of infected drops drastically to 0.18% without deaths in this group. Considering the application of the same number of doses, the curves of infected (**b**) considering Strategies 1 and 2 show that Strategy 2, focused on the immunocompromised individuals, was more efficient in reducing the number of infected and dead. Observing the inset, we notice that Strategy 2 reduced the number of infected by 18.3% and the number of dead by 15.6% about Strategy 1.

The results obtained from Strategy 2 are shown in Figure 5. Figure 5a presents the difference between a scenario without vaccination and a scenario considering the vaccination of 100% of immunocompromised (Strategy 2). Without vaccination, 29.6% of immunocompromised become infected, with the death of 0.59% of the same group. Vaccinating 100% of these individuals, the percentage of infected drops drastically to 0.18% without deaths in this group. Now, considering the application of the same number of doses in each strategy, we show the time evolution of the fraction of infected individuals (Figure 5b) considering Strategies 1 and 2. We observe that Strategy 2 is more efficient in reducing the number of infected and dead individuals. Looking in detail, we note that Strategy 2 reduced the number of infected people by 18.3% and the number of deaths by 15.6% compared to Strategy 1. Similar results were obtained in studies that examined the efficiency of vaccination campaigns using a data-driven epidemic model that incorporates age structure and age-decreasing vaccination policies [38].

In Strategy 2, all immunocompromised individuals were vaccinated. Distributing and applying this same amount of doses randomly among individuals in the lattice (Strategy 1), 24.6% of immunocompromised people were infected, while the mortality rate was 0.5%. Thus, when we analyzed individuals in this group in a scenario in which doses were randomly distributed across the lattice, Strategy 2 showed a 99.3% reduction in the number of infected individuals and a 100% reduction in deaths compared to Strategy 1.

**Figure 6:**
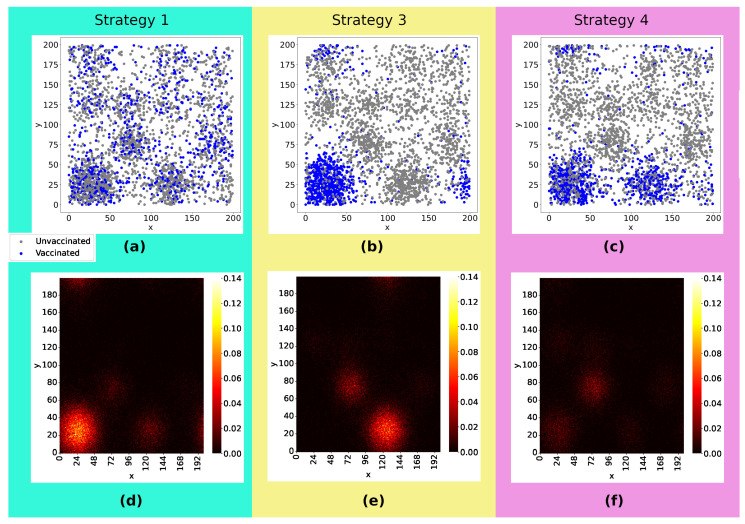
Panels (**a**–**c**) show the initial position of agents, differentiating vaccinated (blue points) from unvaccinated (gray points) individuals. The same amount of doses were applied in the three cases. Panels (**d**–**f**) present heatmaps with the position of each infected individual and the concentration of infected individuals per site 20 weeks after the viral spread has started. These heatmaps were constructed by taking the average of 300 experiments. Comparing the three strategies, we can notice that Strategy 1 was less effective, resulting in a higher concentration of infected individuals in the densest sub-region and allowing the virus to spread further. The number of infected individuals, considering Strategy 3 and 4, is lower than in the first case, with the majority of cases concentrated in the second densest sub-region of the lattice, when the doses are distributed only in the denser sub-region (**e**) and more effective control of viral spread is seen in Strategy 4 (**f**). Rate of vaccination: 10%.

Now, we turn to the effects of the strategies that rely on the spatial distribution of the population. Figure 6a–c illustrates how the vaccines were distributed: In Strategy 1, the doses were equally distributed across the lattice, whereas in Strategy 3, the doses were distributed only in the two denser sub-regions, and in Strategy 4, they are distributed solely in the denser sub-region. The snapshot in Figure 6d–f depicts heatmaps with the sum of infected, dead, and recovered individuals as well as their spatial distribution 20 weeks after the start of viral spread. Comparing the three strategies, we can notice that Strategy 1 was a less effective strategy, with a higher concentration of infected individuals in the densest sub-region and with viruses reaching more regions of the lattice. The number of infected individuals, considering Strategy 3 and 4, is lower than in the first case, with the majority of cases concentrated in the second densest sub-region of the lattice when the doses are distributed only in the denser sub-region (Strategy 3). Strategy 4 emerges as the strategy for best controlling viral spread.

**Figure 7:**
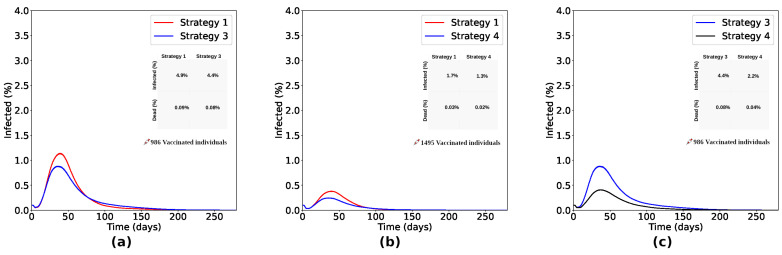
Comparing Strategies 1 and 3, (**a**) the process focusing on vaccinating individuals initially located in the denser sub-region of the lattice (Strategy 3) reduced the number of infected by 8% and the number of deaths by 11% compared with Strategy 1. As shown in (**b**), Strategy 4, which focused on vaccinating individuals initially located in the two denser sub-regions of the lattice, reduced the number of infected by 27.8% and the number of deaths by 33.3% compared with Strategy 1. It shows us that Strategies of vaccinating focused in denser regions are more effective than random distribution (as proposed in Strategy 1). As seen in (**c**), it is important to vaccinate individuals encountered in the denser sub-regions, as well as to consider the neighboring sub-regions, in order to obtain even more efficient results. With the same number of doses, Strategy 4 reduced the number of infected and dead by 50% compared to Strategy 3.

Comparing Strategies 1 and 3 (Figure 7a), the process focusing on the vaccination of individuals initially located in the denser sub-region of the lattice (Strategy 3) reduced the number of infected individuals by 8% and the number of deaths by 11% compared with Strategy 1. As shown in Figure 7b, Strategy 4, which focused on the individuals initially located in the two denser sub-regions of the lattice, reduced the number of infected by 27.8% and the number of deaths by 33.3% compared with Strategy 1. It shows us that vaccination strategies focused on denser regions are more effective than the random distribution (as proposed in Strategy 1). Furthermore, it is important to vaccinate individuals encountered in the denser sub-regions and also consider the neighboring sub-regions to obtain even more efficient results. Comparing Strategies 3 and 4, Strategy 4 reduced the number of infected and dead by 50% compared to Strategy 3, as seen in Figure 7c.

**Figure 8:**
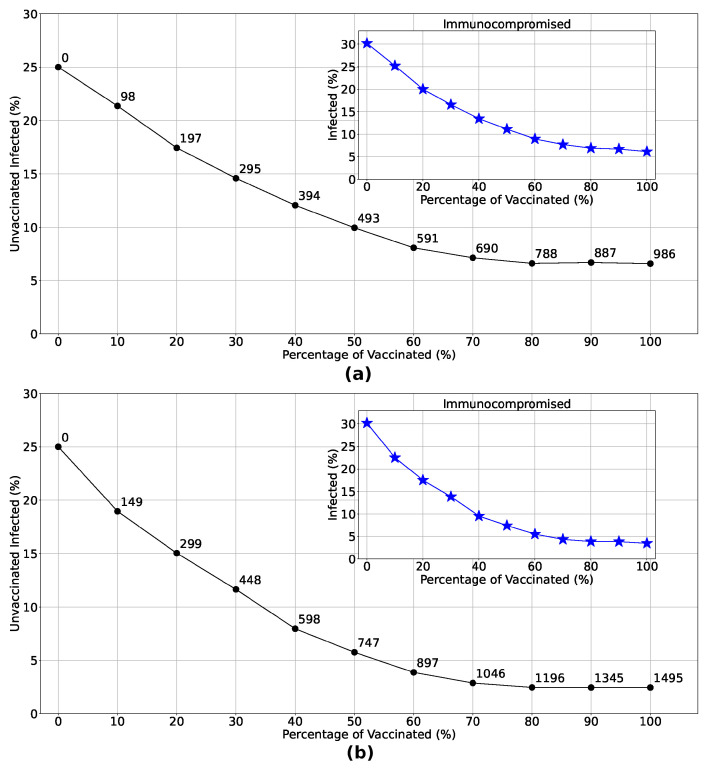
Variation in the percentage of unvaccinated infected individuals with increasing vaccination rates, considering Strategy 3 (Panel (**a**)) and Strategy 4 (Panel (**b**)). Analyzing the number of doses administered in each strategy (numbers highlighted at each point in the main graphs), we see that vaccinating only in the densest region resulted in infection rates for low vaccination percentages very similar to those observed in Strategy 4, given the same number of doses. This further corroborates that focusing on the distribution and administration of vaccines in the most crowded regions is the most efficient strategy in a scenario with few doses available. This is also reflected in the protection of immunocompromised individuals, as seen in the insets.

We will now delve deeper into the impact of vaccination on unvaccinated individuals as vaccination rates increase, considering the spatial strategies. Figure 8 illustrates the exponential decrease in the percentage of unvaccinated infected individuals with the implementation of Strategies 3 and 4. The insets demonstrate a notable reduction in the number of immunocompromised individuals infected when these strategies are employed. Examining the distribution of doses in each strategy (numbers highlighted in the main graphs), it becomes clear that focusing vaccination efforts solely in the most densely populated region yields infection rates comparable to those observed in Strategy 4, particularly when doses are limited. This underscores the effectiveness of prioritizing vaccine distribution and administration in densely populated regions, especially in areas with constrained vaccine availability.

Although Strategies 3 and 4 proved to be more efficient in reducing the number of infected and dead individuals, random vaccination (Strategy 1) is also valuable. It also significantly decreased the number of infected individuals compared to a scenario without vaccines. Furthermore, we reinforce that adopting strategies focused on specific regions can reduce logistical costs and mitigate limitations related to the accessibility, affordability, and acceptability of vaccines, especially in scenarios of low vaccine availability (as occurred with COVID-19, for example).

## 5. Conclusions

We live in an increasingly interconnected society, where interpersonal interactions and our involvement with nature intensify every day. The increase in these interactions could bring humans into contact with previously unknown pathogens. This fierce social activity causes these same pathogens to evolve and acquire different characteristics, thanks to a process called biological pressure. In this sense, society must continue to develop technologies and innovations, developing and improving defense strategies against the actions of these microorganisms.

We saw that the application of the IABM model to study vaccination strategies allows us to analyze scenarios in which a previously vaccinated individual still has a chance of being infected, depending on the conditions imposed on them, as can be seen in Figure 3. The methods used in this model are unprecedented, as a vaccinated individual can become infected depending on the dynamics between their immune system and the invading pathogen. The robustness of the model and its credibility are clear in Figure 4, a scenario where different vaccination percentages are applied. We observed that by increasing the number of inoculated doses, the number of infected and dead people decreases drastically, with no viral spread when at least 70% of the population is previously vaccinated. The Panel shown in Figure 4d shows the effect of herd immunity, with an exponential reduction in the number of unvaccinated infected people in relation to the increase in the vaccination rate, due to the indirect protection generated by those who receive the vaccine.

In order to analyze strategies considering an insufficient number of vaccines for the entire population, we carried out extensive simulations considering four vaccination strategies, as described in Table 1. Considering vaccination of immunocompromised people, we observed a drastic reduction in infection and mortality rates in this group, with a decrease of 18.3% in the number of infected people and 15.6% in the number of deaths compared with Strategy 1, in which a random vaccination process is applied to the general population, as seen in Figure 5.

Strategies targeting spatial disparities were also analyzed, comprising Strategies 3 and 4, whose dose distribution is illustrated in Figure 6b,c. Observing Figure 6d–f, we see that strategies focused on denser regions are more efficient, generating less viral spread and, consequently, fewer infected and dead people. Drawing a parallel between Strategies 3 and 4 and comparing them with Strategy 1, as seen in Figure 7, we see that a vaccination strategy that includes the densest region and its neighborhood (Strategy 4) is more efficient than a strategy that focuses only on the densest region (Strategy 3), reducing the number of infected by 27.8% and the number of deaths by 33.3%, with a reduction of 8% in the number of infected people and 11% in deaths compared to Strategy 1. In a scenario considering a greater quantity of doses available, Strategy 4 reduced the number of infected and dead people by 50% when compared to Strategy 3. In another aspect, when the number of available doses is smaller, we observe in Figure 8 that the number of unvaccinated infected people and the infection rate of immunocompromised people are very close in both strategies, corroborating that, in a scenario of scarce doses, the most efficient strategy should focus on sub-regions with larger agglomerations. In addition to reducing the percentage of infected people observed in vaccination strategies focused on specific sub-regions, these strategies are also beneficial when considering logistical costs, storage and transportation of doses, and limitations related to the accessibility, affordability, and acceptability of vaccines.

Defining increasingly efficient public policies is extremely necessary. In an interconnected society, with complex challenges and the imminent occurrence of new outbreaks, epidemics, and pandemics, it is mandatory to invest in the development of robust models and techniques that provide knowledge and methods that make human life safer, to reduce deaths, economic costs, and expenses associated with logistics. By doing so, we can help ensure the survival of our society in the face of an active process of disease spread. In a more specific context, we must develop solid and efficient vaccination strategies, taking into account the indirect protection of those who, for some reason, cannot be vaccinated.

## Figures and Tables

**Table 1:** Description of the group(s) vaccinated in each vaccination strategy.

Strategy	Vaccinated Group
Strategy 1	General population, not considering spatial and individual’s characteristics (random vaccination).
Strategy 2	Immunocompromised individuals.
Strategy 3	Individuals initially located in the denser sub-region of the lattice.
Strategy 4	Individuals initially located in the two denser sub-regions of the lattice.

## Data Availability

No data were used for the research described in the article.

## Abbreviations

The following abbreviations are used in this manuscript:

ABM	Agent-based Model
IABM	Immunity Agent-Based Model
Ax	Asymptomatic
Sx	Symptomatic
SEIR	Susceptible-Exposed-Infected-Recovered
SVEIR	Susceptible-Vaccinated-Exposed-Infected-Recovered
